# The characteristics of brain network in patient with post-stroke depression under cognitive task condition

**DOI:** 10.3389/fnins.2023.1242543

**Published:** 2023-08-16

**Authors:** Yu Peng, Yang Zheng, Ziwen Yuan, Jing Guo, Chunyang Fan, Chenxi Li, Jingyuan Deng, Siming Song, Jin Qiao, Jue Wang

**Affiliations:** ^1^The Key Laboratory of Biomedical Information Engineering of Ministry of Education, School of Life Sciences and Technology, Institute of Biomedical Engineering, Xi’an Jiaotong University, Xi’an, China; ^2^Department of Rehabilitation, First Affiliated Hospital of Xi’an Jiaotong University, Xi’an, China; ^3^The State Key Laboratory for Manufacturing Systems Engineering, School of Mechanical Engineering, Institute of Engineering and Medicine Interdisciplinary Studies, Xi’an Jiaotong University, Xi’an, China; ^4^Department of Military Medical Psychology, Air Force Medical University, Xi’an, China

**Keywords:** post-stroke depression, cognitive task, brain network, fNIRS, graph theory

## Abstract

**Objectives:**

Post-stroke depression (PSD) may be associated with the altered brain network property. This study aimed at exploring the brain network characteristics of PSD under the classic cognitive task, i.e., the oddball task, in order to promote our understanding of the pathogenesis and the diagnosis of PSD.

**Methods:**

Nineteen stroke survivors with PSD and 18 stroke survivors with no PSD (non-PSD) were recruited. The functional near-infrared spectroscopy (fNIRS) covering the dorsolateral prefrontal cortex was recorded during the oddball task state and the resting state. The brain network characteristics were extracted using the graph theory and compared between the PSD and the non-PSD subjects. In addition, the classification performance between the PSD and non-PSD subjects was evaluated using features in the resting and the task state, respectively.

**Results:**

Compared with the resting state, more brain network characteristics in the task state showed significant differences between the PSD and non-PSD groups, resulting in better classification performance. In the task state, the assortativity, clustering coefficient, characteristic path length, and local efficiency of the PSD subjects was larger compared with the non-PSD subjects while the global efficiency of the PSD subjects was smaller than that of the non-PSD subjects.

**Conclusion:**

The altered brain network properties associated with PSD in the cognitive task state were more distinct compared with the resting state, and the ability of the brain network to resist attack and transmit information was reduced in PSD patients in the task state.

**Significance:**

This study demonstrated the feasibility and superiority of investigating brain network properties in the task state for the exploration of the pathogenesis and new diagnosis methods for PSD.

## 1. Introduction

Post-stroke depression (PSD) is the most common neuropsychiatric complication after stroke (Taylor-Rowan et al., 2019). About one-third of stroke survivors suffer from PSD (Guo et al., 2022), which has a significant impact on their rehabilitation outcomes and quality of life (Paolucci, 2017). It has been demonstrated that early diagnosis, prevention and treatment of PSD are very important for stroke survivors (Koyanagi et al., 2021). However, the pathogenesis of PSD is still being investigated (Guo et al., 2022). Currently, the diagnosis of PSD in clinics mainly relies on the subjective scale-based evaluation of patients’ emotion state and an objective indicator is urgently needed.

The combination of the modern brain imaging technology and the complex network theory, i.e., the graph theory provides a powerful tool to analyze the human brain networks (Power et al., 2010; Wang and Wang, 2014). The study of brain functional networks provides a new perspective for understanding the pathological mechanism and then the assistance for the early diagnosis of neuropsychiatric diseases (Wang et al., 2021). Previous studies have suggested that PSD might be caused by the damage to some specific brain network (Boes et al., 2015). Zhang et al. (2019) scanned the amygdala in the affective network using the functional magnetic resonance imaging (fMRI) to study the characteristics of the brain functional network in the PSD patients with left temporal lobe infarction in the resting state. They found that PSD was closely related to the reorganization of the damaged brain networks mainly involving the amygdala and the prefrontal cortex. Similarly, Shi et al. (2017) collected the fMRI data from the cingulate cortex in the resting state and compared the topological properties of the default mode network (DMN) between the stroke survivors with and without PSD. The results showed that the functional connectivity of the anterior cingulate cortex with the prefrontal cortex, cingulate cortex, and motor cortex in PSD patients was significantly reduced. However, the functional connectivity of the anterior cingulate cortex with the hippocampus, parahippocampal gyrus, insula and amygdala was enhanced. These indicated that the pathogenesis of PSD was possibly related to the altered connectivity in the DMN. Balaev et al. (2018) further demonstrated that both the DMN and the salience network were changed in the PSD patients. In another resting-state fMRI study, Egorova et al. (2018) found that the functional connectivity between the left dorsolateral prefrontal cortex and the right superior limbic gyrus in the PSD patients was significantly reduced, and the decline of the connectivity in the frontoparietal cognition control network was positively correlated with the severity of depression. In general, these studies found abnormal brain network connectivity at the prefrontal cortex, amygdala or hippocampus regions in the PSD patients. However, the brain network properties in the resting state were investigated the most, and furthermore their results were inconclusive for the cause of PSD from the view of altered brain functional networks.

Studies have shown that the pattern of brain functional connectivity in the task state was different from that in the resting state at both the neuron and system levels (Zhang et al., 2010; Cole et al., 2014; Gerchen et al., 2014; Foster et al., 2015). With most resting-state networks still being identifiable in the task state (Cole et al., 2014; Krienen et al., 2014), the differences of the brain network connectivity between the resting and task conditions observed using the noninvasive functional neuroimaging techniques at the system level might be subtle. However, these differences are widely distributed across the brain. For example, based on the Human Connectome Project (HCP) task set, up to 38% of the connectivity were significantly different between the task and resting states (Barch et al., 2013; Cole et al., 2014). Kaufmann et al. (2017) recently reported that 76.2% of the connectivity were different across 6 tasks. Although the brain network topology may remain unchanged overall, the network functional connectivity indeed reconfigured in some ways when switching from the resting to the task state (Cole et al., 2014; Krienen et al., 2014). For example, the brain became less segregated and the functional connectivity was more stable in the task state (Di et al., 2013). In addition, both the hub location and the communication frequency can be modulated by the participation of tasks. Therefore, compared with the resting state, it might provide us another alternative to explore the pathogenesis of PSD by investigating the brain network in the task state, due to its widely varied and more stable brain network connectivity.

Facial emotion recognition is one of the most commonly used tasks in the research of depression (Fusar-Poli et al., 2009), mainly involving two types of experiment paradigms. One is the explicit task that requires the subjects to judge the emotion categories, which is mostly used to study the emotional dysfunction in subjects with depression. The other one is the implicit task that requires the subjects to judge the gender with the presentation of different emotional expressions. This paradigm is typically used to estimate the ability of the brain to process emotional faces unconsciously. Via the facial emotion recognition tasks, researchers have obtained a certain understanding of the neural mechanism for the negative cognitive processing in patients with depression. However, there are some limitations in the facial emotion recognition tasks. Firstly, the expression pictures come from different countries, thus making it difficult to avoid the interference of race, age etc. Secondly, people from different countries with different cultures may have different degrees of recognition of the same expressions. Lastly, the gender might also affect the recognition of facial expressions because women generally have richer emotions and are more sensitive to expressions (Gard and Kring, 2007; Jenkins et al., 2018). Compared with the facial emotion recognition task, the classic ‘oddball’ task paradigm that is widely used in the event-related potential studies has no such limitations. For example, the P300 wave elicited in the oddball task is considered to be an endogenous evoked potential related to the cognitive function of brain (Polich, 2007). As a potential reliable biomarker for the advanced cognitive functions such as the attention and working memory (van Dinteren et al., 2014), the P300 wave has been widely used for the assessment of cognitive disorders. It is commonly believed that there is a close relationship between the cognition and emotion. For example, early PSD aggravates the cognitive impairments in elderly male stroke patients (Shin et al., 2022). In addition, the impairment of working memory was believed to be an important indicator of the cognitive impairment in depression. Meanwhile, researchers have found that the P300 wave was a reliable psychological measurement in both depression and healthy individuals (Klawohn et al., 2020). Based on these research findings, it was hypothesized in this study that the oddball task, as a classic experiment paradigm for cognitive function assessing, may possibly get the damaged brain network associated with PSD involved and thereby, help obtain the brain network characteristics that can reflect the neural mechanism of PSD. Meanwhile, as far as we know, no previous studies have used the oddball task paradigm to investigate the brain network property of PSD.

Most previous studies on the brain network of PSD utilized the fMRI data to measure the metabolic activities in brain. As another non-invasive brain functional imaging technique, the functional near-infrared spectroscopy (fNIRS) has the advantages of low cost, portability, and convenience for a variety of tasks (Ferrari and Quaresima, 2012; Hong and Khan, 2017) and more importantly, it has a higher temporal resolution than fMRI. Therefore, it has been applied in different clinical settings, especially in the field of neuroscience. Previous studies have shown that the oxyhemoglobin concentration (HbO) measured by fNIRS may be a useful tool for diagnosing PSD (Koyanagi et al., 2021). However, to the best of our knowledge, there is still a lack of studies that use the fNIRS signals to analyze the brain network of PSD patients.

In this study, in order to investigate the altered brain network characteristics of PSD in the task state and as a result, obtain the possible biomarkers for PSD diagnosis, the oddball task paradigm was performed by stroke survivors with and without PSD (non-PSD). The fNIRS data were collected under both the task and resting conditions, and the brain functional connectivity and network properties based on the graph theory were analyzed and compared between the PSD and non-PSD patients. Based on the previous studies, it was hypothesized that some of the altered brain network properties that accounts for PSD might be manifested in the task state other than the resting state, resulting in more distinct brain network connectivity patterns and characteristics in the cognitive task state compared with the resting state. Our results possibly provide a new perspective to explore the pathogeneses and new diagnosis methods for PSD.

## 2. Materials and methods

### 2.1. Subjects

All recruited subjects were post-stroke patients undergoing the rehabilitation therapy at the Rehabilitation Department of the First Affiliated Hospital of Xi’an Jiaotong University from June 2022 to November 2022. The inclusion criteria were as follows: (1) 30–85 years old; (2) stroke confirmed by computed tomography or magnetic resonance imaging; (3) first-ever stroke with the onset within 1–12 months from then; (4) consciousness with the ability to finish the experiment task. The exclusion criteria were: (1) history of mental illness, such as schizophrenia, mood disorders; (2) substance abuse; (3) severe neurological impairment, such as hearing impairment and physical weakness; (4) metal implants in the brain, such as deep brain stimulators; (5) cranioplasty. This study was approved by the Ethics Committee of the First Affiliated Hospital of Xi’an Jiaotong University on March 27, 2021 (approval number: XJTU1AF2023LSK-2021- 175), and all patients or their authorized representatives signed the informed consent.

All subjects were assessed using the Hamilton Depression Scale with 24 items (HAMD) that has been widely used in the diagnosis and severity assessment of depression, and higher scores indicate more severe depression. The assessment was performed by 2 trained psychiatrists in the hospital. All recruited subjects were divided into 2 groups based on their HAMD scores. The subjects with the score higher than or equal to 8 constituted the PSD group. The other subjects with scores less than 8 constituted the non-PSD group. In addition, the Montreal Cognitive Assessment Test (MoCA) and the Mini-mental State Examination (MMSE) were performed to assess the cognitive state of subjects.

### 2.2. Experiment procedure

#### 2.2.1. Oddball task paradigm

**Figure 1:**
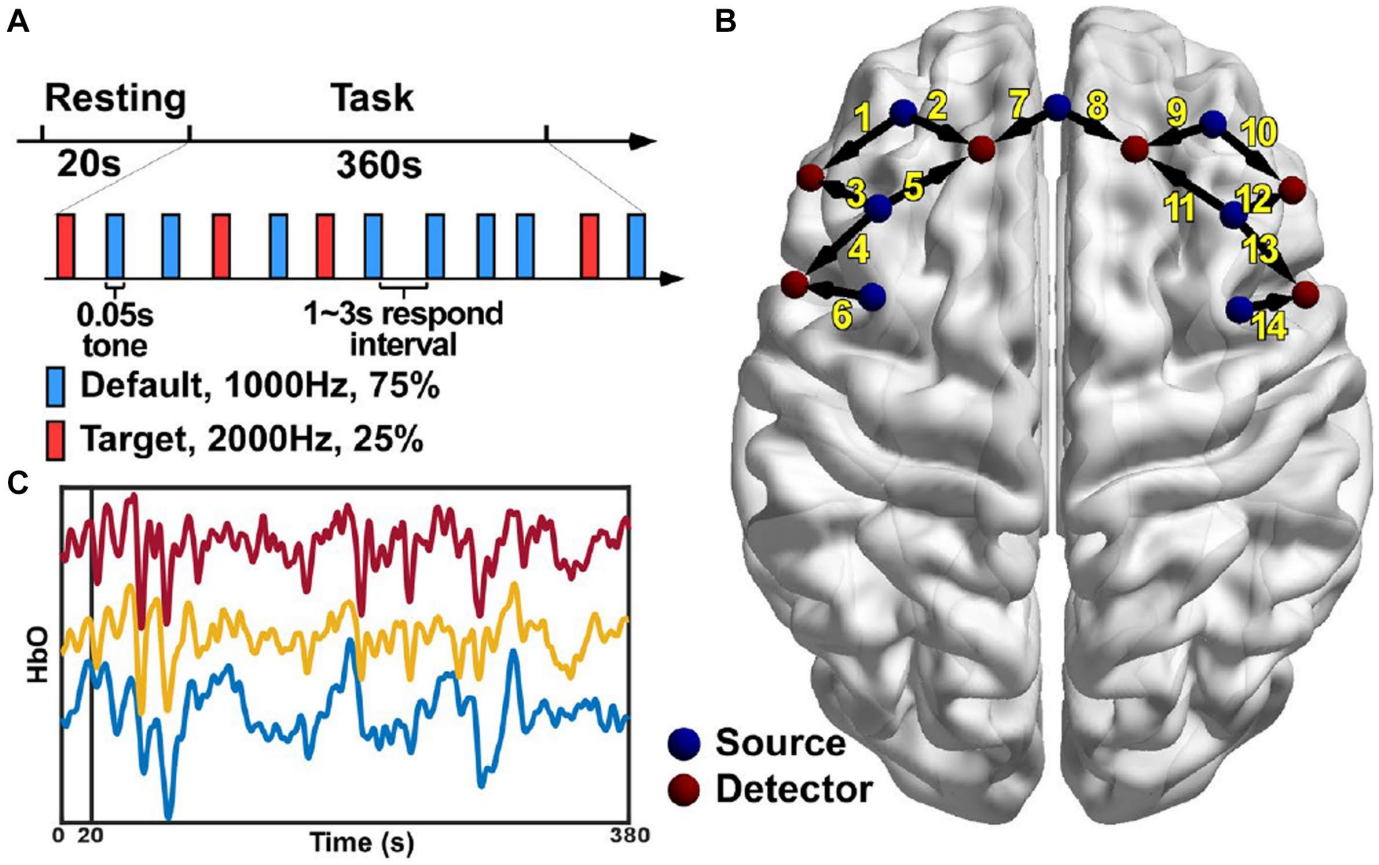
Experiment paradigm **(A)**, the locations of the 13 optodes including 7 sources and 6 detectors **(B)** and the HbO signals of three channels from a representative subject **(C)**.

The classic oddball paradigm was used in this study. The subjects were presented with two kinds of auditory stimuli both at the intensity of 85 dB. The default or the non-deviant stimulus was a low-frequency tone at 1,000 Hz while the target or the deviant stimulus was a high-frequency tone at 2,000 Hz (Figure 1A). Each stimulus lasted for 0.05 s and there was a random respond interval lasting 1 ~ 3 s between two adjacent stimuli. The total stimulation or task period lasted 360 s with 25% as the deviant stimuli and 75% as the non-deviant stimuli. The subjects were requested to press the button using their thumbs of the unparalyzed side immediately after the deviant sound was heard. Before the task period, there was a 20-s resting period served as the baseline. The subjects were sat comfortably with their eyes closed and their bodies relaxed during the whole experiment, and asked to concentrated their attention on the auditory stimuli during the stimulation period.

#### 2.2.2. Data recording

The NirScan-6000A system (Huichuang Medical Equipment Co., Ltd., Danyang, China) was used to record the fNIRS signals in this study. The wavelength of the near-infrared light used in this system was 730, 808 and 850 nm, respectively. Thirteen optodes were used including 7 light sources and 6 light detectors with an inter-optode distance of 3 cm. This optode configuration resulted in 14 channels of fNIRS signals in total covering the left and the right dorsolateral prefrontal (DLPF) cortex, respectively (Figure 1B). The sampling rate was 11 Hz and data recording was performed in a quiet and dark room.

### 2.3. Data processing

#### 2.3.1. Preprocessing

The original light intensity signal was converted into the optical density (OD) signal for individual channels. Then, an automatic motion artifact removal procedure was performed using a sliding window method for individual channels. Specifically, within each 0.5-s window, if the difference between the maximum and the minimum of the OD signal was more than 6 times the standard deviation of the whole trial, the window was considered to contain motion artifacts and the corresponding OD signal was discarded and then reconstructed using the spline interpolation method. The OD signal was then filtered using a band-pass filter between 0.01 and 0.2 Hz to remove the components from the heart rate, the blood pressure and the respiratory activity. Lastly, the processed OD signal was converted into the HbO signal (Figure 1C) based on the modified Beer–Lambert law. The preprocessing was performed using the analysis program NirSpark (Danyang Huichuang Medical Equipment Co., Ltd., China).

#### 2.3.2. Brain network characteristics

Pearson correlation coefficients between the HbO signals of all possible pairs of channels were calculated using the data from the task and the resting period respectively, yielding two 14 × 14 connectivity matrices for each subject. The Fisher-z transformation was then performed on the connectivity matrices. In this study, the GRETNA software (Wang et al., 2015) was used to estimate the brain network metrics according to the graph theory. Specifically, only positive connectivity was considered in this study by setting the negative matrix entries to zeros, and the connectivity matrices were then binarized using the sparsity threshold from 0.15 to 0.5 at intervals of 0.05. The threshold value was defined as the ratio of the number of retained edges divided by the maximum possible number of edges in the network. The global metrics including the small-world parameters [clustering coefficients (Cp), Gamma, Lambda, characteristic path length (Lp), Sigma], the local efficiency (Eloc), the global efficiency (Eg), the assortativity (r), and the hierarchy (b) were calculated. And the estimated nodal metrics included the nodal clustering coefficient (NCp), the nodal efficiency (Ne), the nodal local efficiency (NLe), the degree centrality (Dc), and the betweenness centrality (Bc). Since different metric values can be obtained under individual thresholds, the area under the curve (AUC) for each network metric was calculated for further analysis.

#### 2.3.3. Classification between PSD and non-PSD patients

The diagnosis of a disease can be eventually translated into a classification problem. In this study, in order to verify whether the PSD and non-PSD patients can be distinguished using the extracted brain network characteristics, they were grouped together as the features and the cost-sensitive support vector machine (Luts et al., 2010) was used as the classifier that has been used in our previous studies (Zheng et al., 2016; Zheng and Xu, 2019). The classification accuracy defined as the ratio between the number of correctly identified patients and the total number of patients was used as the performance measurement. In order to avoid the in-sample optimization problem, the 8-fold cross-validation procedure was performed to evaluate the classification accuracy under the task and the resting condition, respectively.

#### 2.3.4. Statistical analysis

The statistical analysis was performed using the SPSS (IBM SPSS Statistics for Windows, version 22.0, IBM Corp.). All comparison were performed between the PSD and non-PSD groups. Specifically, the demographic information was compared using the two-tail two-samples t-test for the measurement data and the Fisher’s exact test for the categorical data. The connectivity strength and the brain network characteristics were compared using the one-tail two-samples t-test. The normality was verify using the Shapiro–Wilk test. If the normality was not satisfied, the Kruskal-Wallis rank sum test was then used. The significant level was set at *p* < 0.05.

## 3. Results

### 3.1. Demographic information

The basic demographic and clinical information of the recruited subjects is listed in Table 1. The statistical analysis results showed that there was no significant difference in the age, gender and the education level between the PSD and the non-PSD group. As for the stoke type, lesion location, the time after stroke onset and the available hand to press the button, there was no significant difference between the two groups either. The HAMD score of the PSD group was significantly higher than that of the non-PSD group, while the MMSE and MoCA scores that mainly reflected the cognitive state showed no significant differences.

**Table 1:** Demographic and clinical information of subjects.

	PSD	Non-PSD	*p-*value
Subject no.	19	18	–
Age (years)	65.8 ± 8.7	59.1 ± 13.6	0.077^a^
Gender (M/F)	13/6	15/3	0.447^b^
Education (≤6 years)^d^	2	0	0.486^b^
Time after onset (days)	81 (32.25–97.5)	67.5 (32–142)	0.682^c^
Manipulating hand (R/L)	12/7	8/10	0.330^b^
Stroke type (I/H)	15/4	11/7	0.295^b^
Lesion location (SC/C)	18/1	14/4	0.180^b^
MMSE score	23.1 ± 4.1	23.6 ± 4.8	0.707^a^
MoCA score	16.4 ± 5.5	18.6 ± 5.7	0.243^a^
HAMD score	15.0 (12.0–17.5)	2.5 (1.0–4.8)	0.001^c^

Values are presented as number of patients, mean ± SD, or median (Q1–Q3).

I, ischemic; H, hemorrhagic; SC, subcortex; C, cortex.

^a^T-test. ^b^Fisher’s exact test. ^c^Kruskal-Wallis rank sum test. ^d^The number of subjects with less than 6 years of education.

### 3.2. Functional connectivity strength

**Figure 2:**
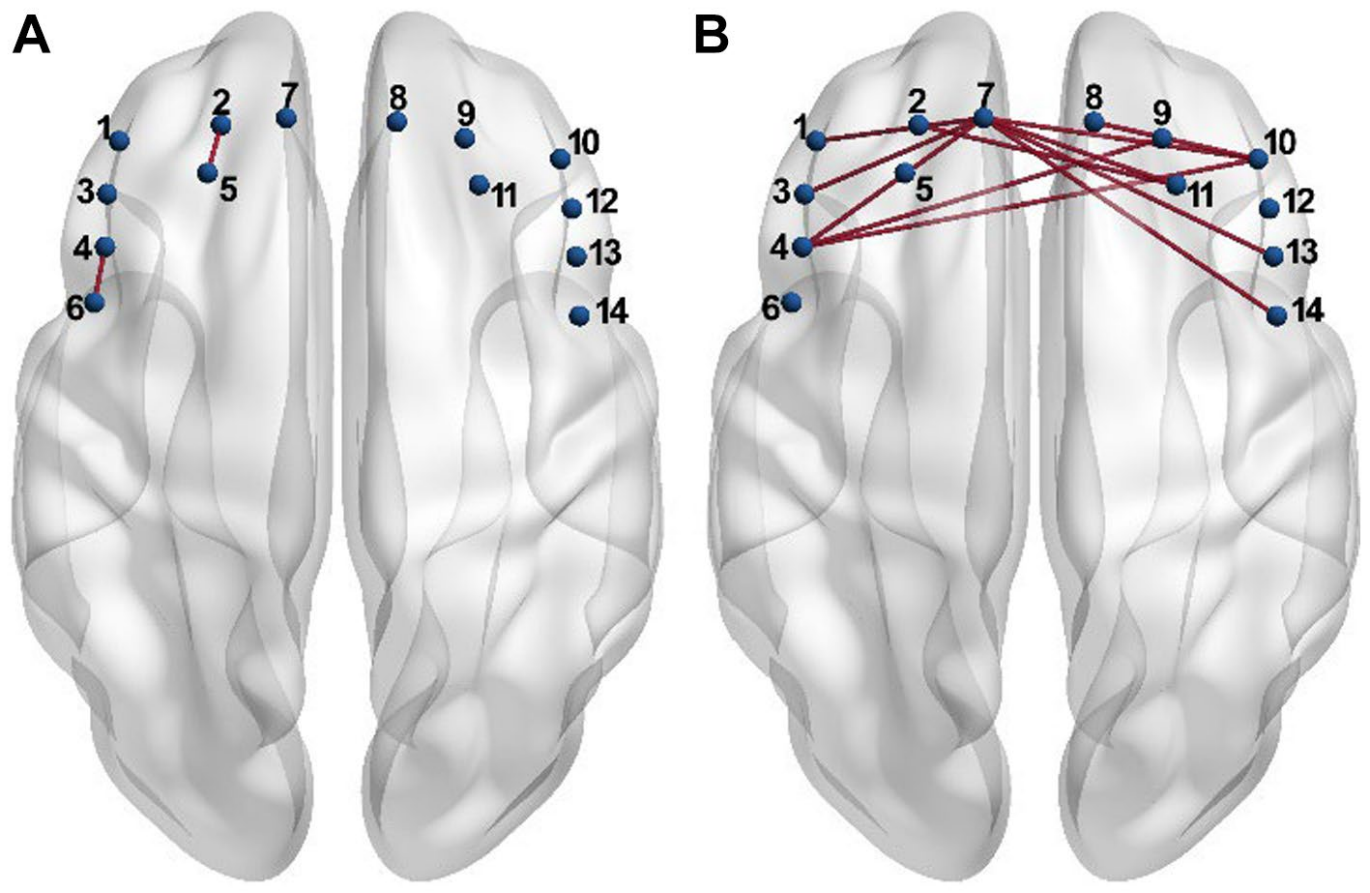
The connectivity showing significant differences between the PSD and the non-PSD groups in the resting **(A)** and the task **(B)** state, respectively.

Each entry in the connectivity matrix quantifies the interaction strength of the HbO signals between two brain regions covered by the two corresponding channels separately. In order to investigate whether the connectivity strength was altered by the PSD, the connectivity matrix was compared entry-by-entry between the two groups under the resting and task conditions, respectively. The statistical analysis results showed that the connectivity between channel 4 and 6, and between channel 2 and 5 for the PSD group was significantly higher than the corresponding connectivity strength for the non-PSD group in the resting state (*p* < 0.05) (Figure 2A). Compared with the resting state, more connectivity showed significant differences between the two groups in the task state (Figure 2B). In contrast to the resting state, the connectivity strength reduced significantly for the PSD group compared with the non-PSD group (*p* < 0.05). For example, the most evident difference was that the connectivity between the middle front area (channel 7) and the lateral areas (channel 1, 3, 4, 10, 11, 13, and 14) was reduced for the PSD subjects compared with the non-PSD subjects in the task state.

### 3.3. Brain network characteristics

#### 3.3.1. Global metrics

**Figure 3:**
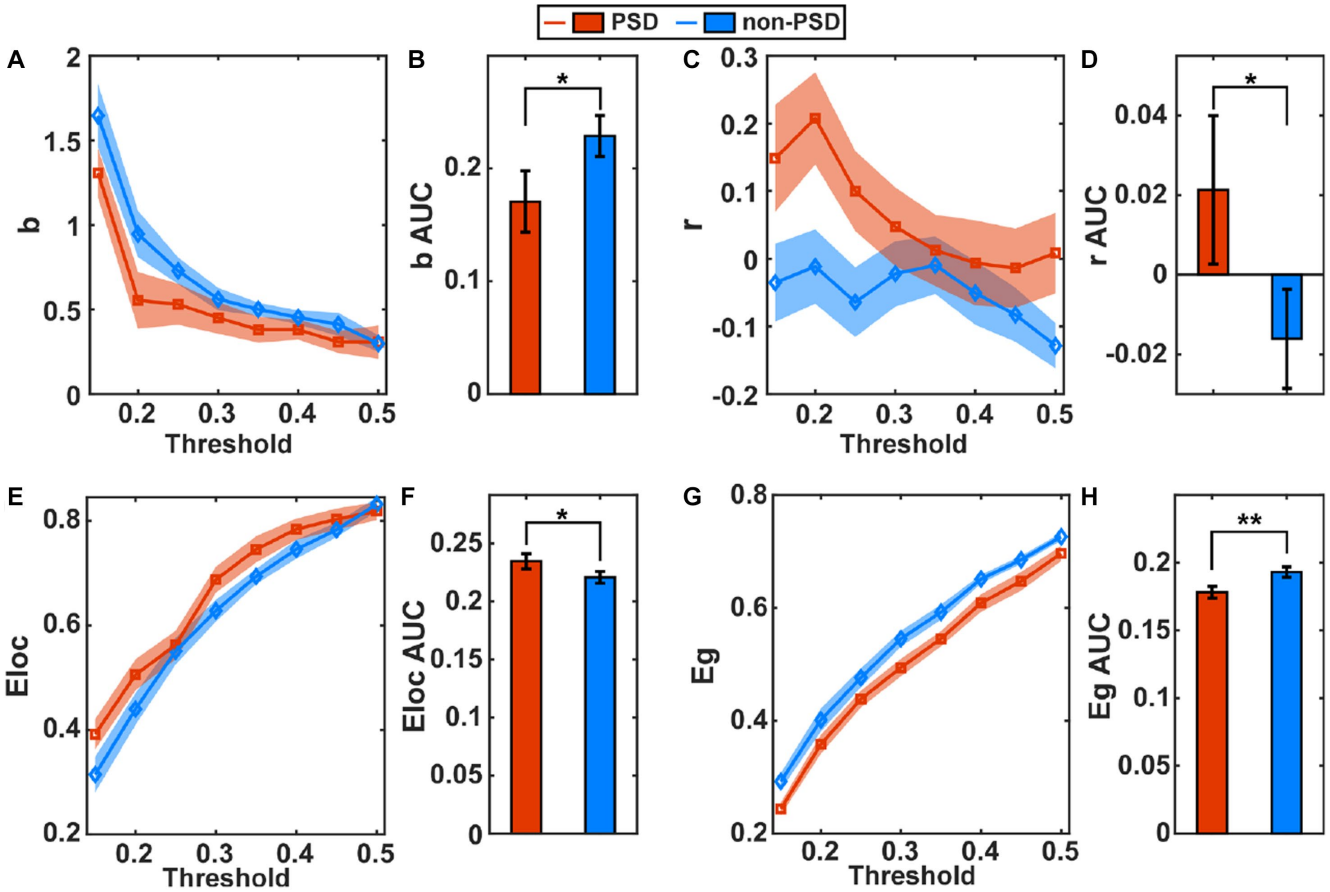
The average of the global metric hierarchy **(A)**, assortativity **(C)**, local efficiency **(E)** and global efficiency **(G)** for individual thresholds under the task condition across subjects. The shadow area represents the standard error. The comparison of the AUC of the metric hierarchy **(B)**, assortativity **(D)**, local efficiency **(F),** and global efficiency **(H)** between the PSD and the non-PSD group. The error bars represent the standard error. *p < 0.05, **p < 0.01.

The estimated global metrics including the hierarchy (b), assortativity (r), local efficiency (Eloc), and global efficiency (Eg) in the task state are compared in Figures 3A,C,E,G, respectively, between the PSD and the non-PSD group. The AUC of the four metrics across different thresholds were then estimated for individual subjects and compared between the two groups (Figures 3B,D,F,H). The results showed that the hierarchy (*p* < 0.05) and the global efficiency (*p* < 0.01) of the PSD group was significantly lower than that of the non-PSD group. On the contrary, the assortativity and the local efficiency of the PSD group were significantly higher than that of the non-PSD group (*p* < 0.05). These four metrics in the resting state were also compared between the PSD and the non-PSD group. However, none of them showed significant differences (*p* > 0.05).

**Figure 4:**
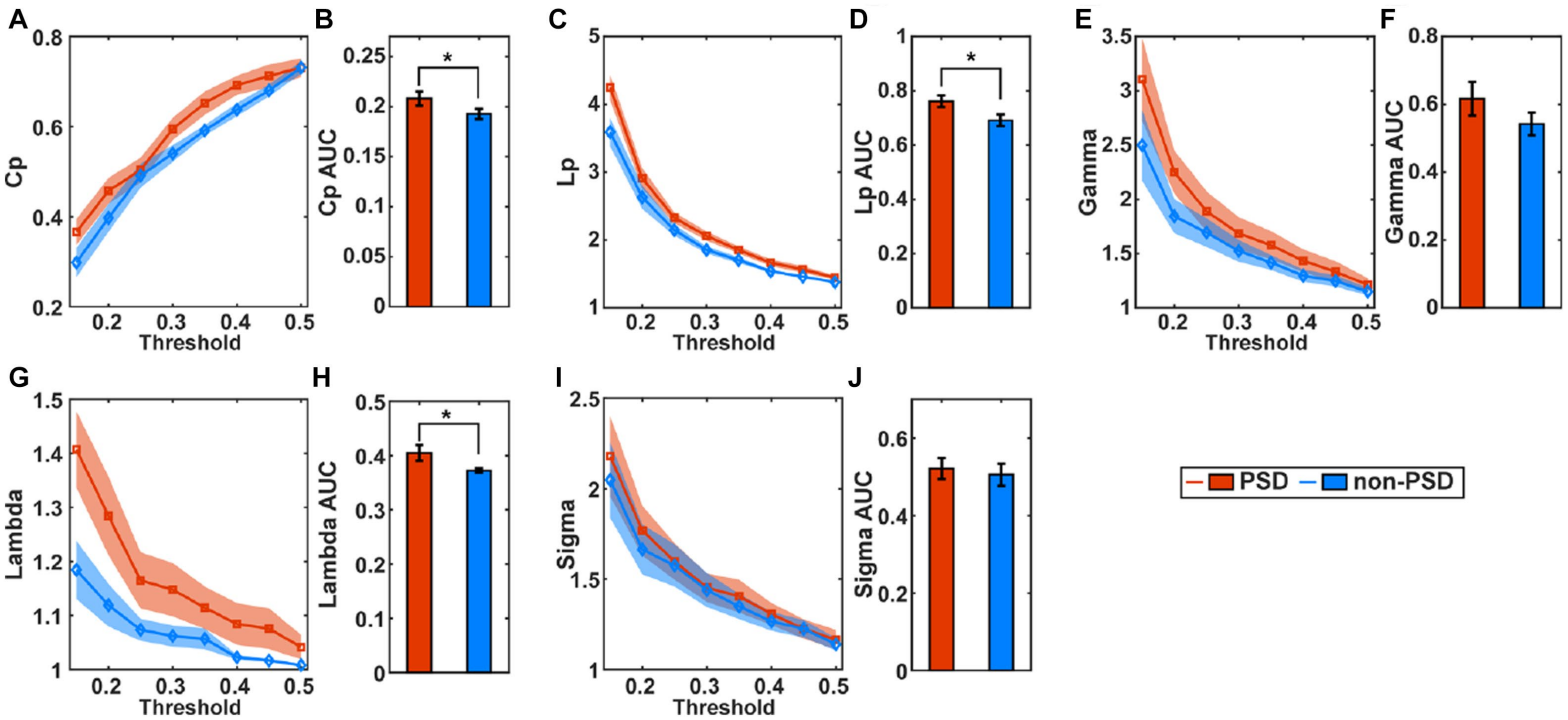
The average of the small-world parameters including the clustering coefficients (Cp), **(A)**, characteristic path length **(C)**, Gamma **(E)**, Lambda **(G),** and Sigma **(I)** for individual thresholds in the task state across subjects. The shadow area represents the standard error. The comparison of the AUC of the small-world parameters Cp **(B)**, Lp **(D)**, Gamma **(F)**, Lambda **(H),** and Sigma **(J)** between the PSD and the non-PSD group. The error bars represent the standard error. *p < 0.05.

Figure 4 compares the small-world properties between the PSD group and the non-PSD group in the task state. The average of the clustering coefficients (Cp), characteristic path length (Lp), Gamma, and Lambda across subjects from the PSD group was higher than that from the non-PSD group (Figures 4A,C,E,G) for all thresholds while the metric Sigma was comparable between the two groups (Figure 4I). The further statistical analysis using the two-samples *t*-test of the AUC of individual metrics showed that the metric Cp, Lp and Lambda of the PSD group was significantly higher than that of the non-PSD group (*p* < 0.05) while there was no significant differences of the metric Gamma and Sigma (p > 0.05). These small-world parameters in the resting state were also compared between the two groups. However, none of these small-world parameters showed significant differences between the two groups (*p* > 0.05).

#### 3.3.2. Nodal metrics

**Figure 5:**
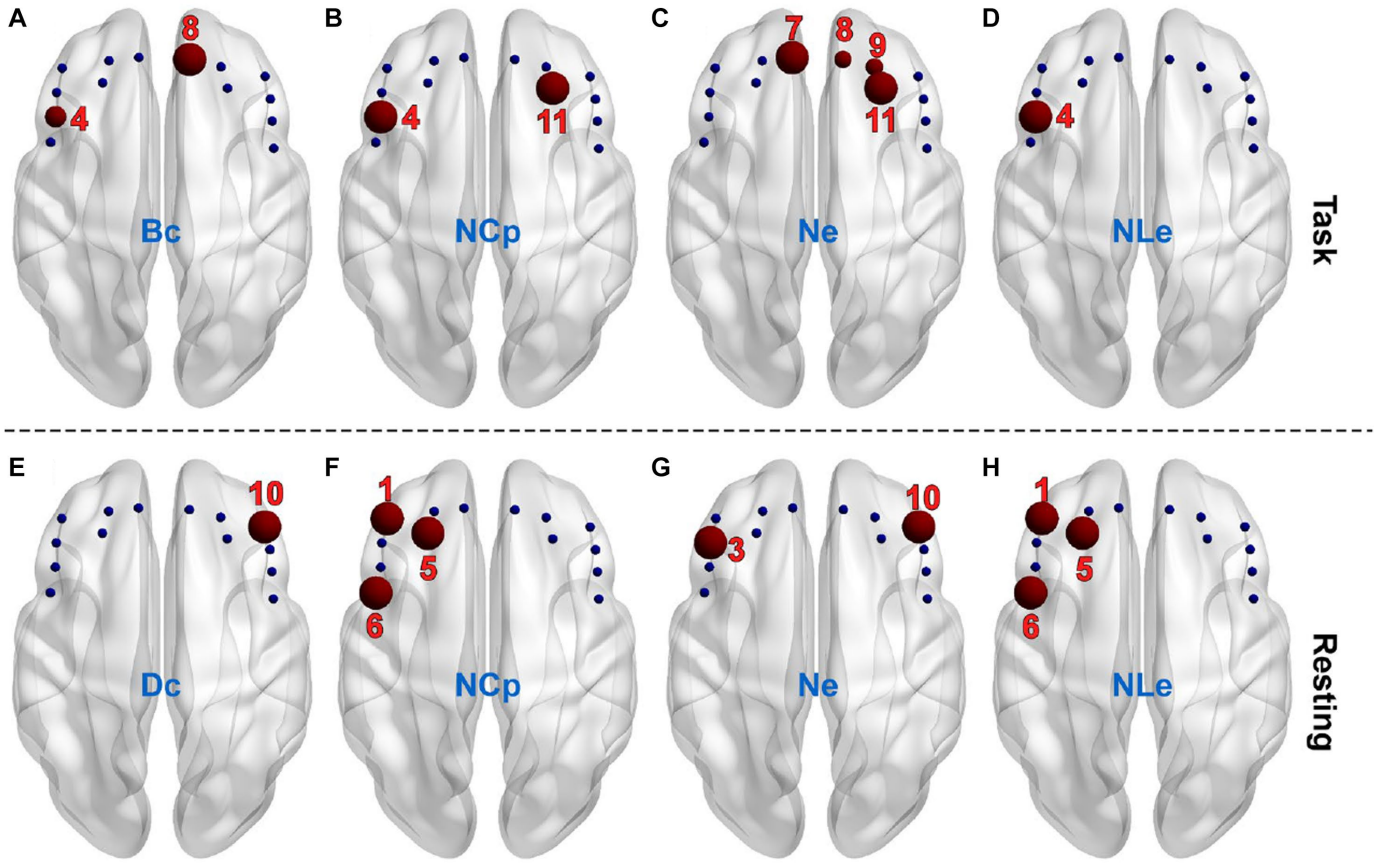
The channels (red balls) showing significant differences between the PSD and the non-PSD groups for the metric betweenness centrality **(A)**, nodal clustering coefficient **(B)**, nodal efficiency **(C)** and nodal local efficiency **(D)**, respectively in the task state. The channels showing significant differences between the PSD and the non-PSD groups for the metric degree centrality **(E)**, nodal clustering coefficient **(F)**, nodal efficiency **(G)** and nodal local efficiency **(H)**, respectively in the resting state.

The AUC of individual nodal metrics were fist calculated across thresholds for individual channels and individual subjects and then compared between the PSD and the non-PSD group channel-by-channel using the two-samples t-test. In the task state, the betweenness centrality (Bc) in channel 4 and 8 for the PSD group was significantly smaller compared with the non-PSD group (*p* < 0.05) (Figure 5A). The nodal efficiency (Ne) in channel 7, 8, 9, and 11 for the PSD group was also significantly reduced compared with the non-PSD group (channel 8 and 9, *p* < 0.05; channel 7 and 11, *p* < 0.01) (Figure 5C). In contrast to the above two metrics, the nodal clustering coefficient (NCp) of channel 4 and 11 (Figure 5B), and the nodal local efficiency (NLe) of channel 4 (Figure 5D) for the PSD group was significantly higher than that for the non-PSD group (*p* < 0.05).

Compared with the task state, the variation of the nodal metrics between the two groups in the restring state shows different patterns. Firstly, the betweenness centrality (Bc) showed no significant difference in either of the 14 channels. Instead, the degree centrality (Dc) of channel 10 for the PSD group was significantly lower compared with the non-PSD group (*p* < 0.05) (Figure 5E). Secondly, even though there were also significant differences of the nodal clustering coefficient (NCp), the nodal efficiency (Ne) and the nodal local efficiency (NLe) between the two groups, the channels that showed significant differences were different from the task state. Specifically, the nodal clustering coefficient (Figure 5F) and the nodal local efficiency (Figure 5H) of channel 1, 5, and 6 for the PSD group were significantly higher compared with the non-PSD group (*p* < 0.05), and the nodal efficiency (Figure 5G) of channel 3 and 10 for the PSD group was significantly reduced compared with the non-PSD group (*p* < 0.05).

#### 3.3.3. Correlation with HAMD scores

**Figure 6:**
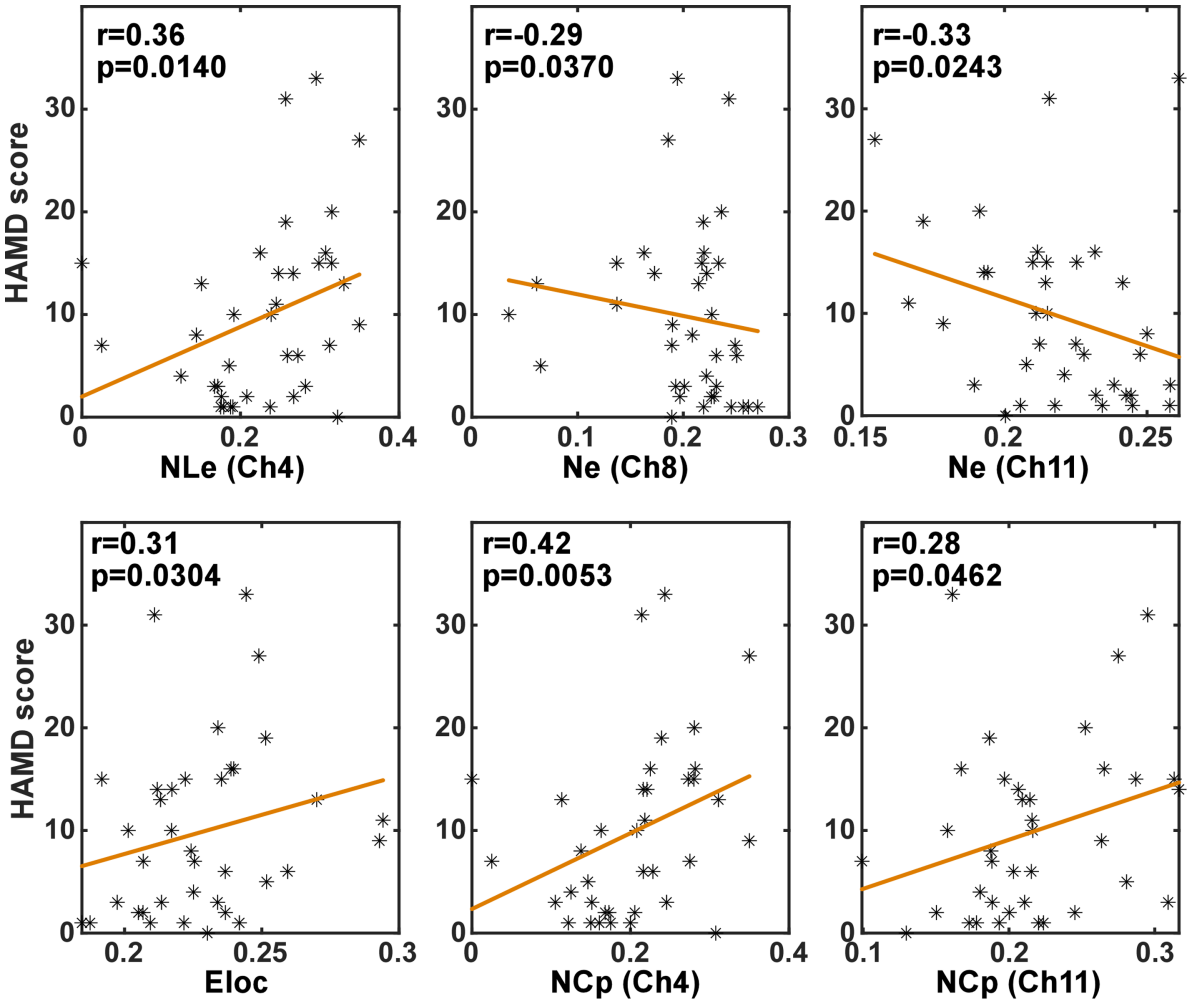
The results of the correlation analysis between the brain network characteristics and the HADM scores in the task state.

**Figure 7:**
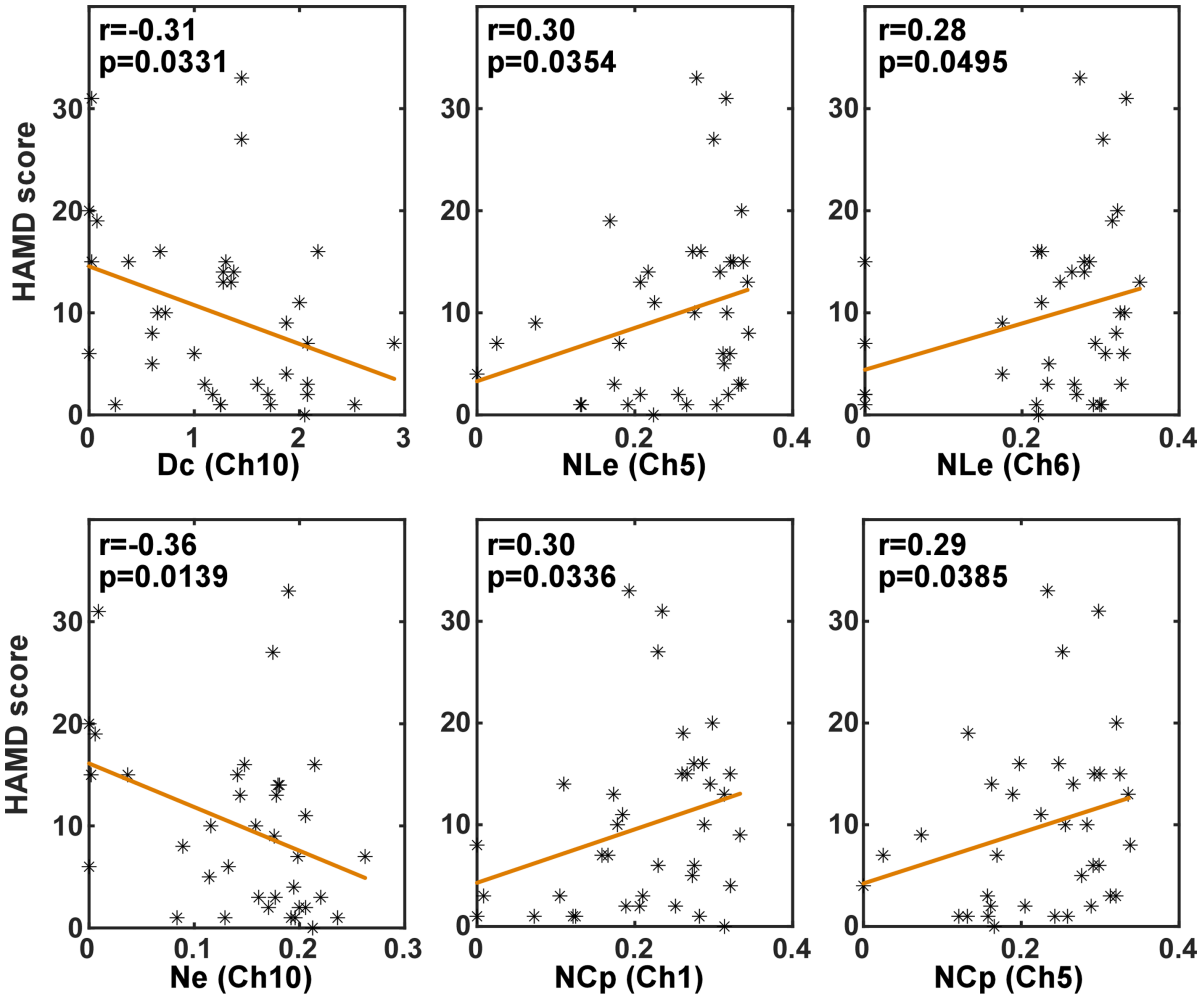
The results of the correlation analysis between the brain network characteristics and the HADM scores in the resting state.

In order to investigate whether the brain network characteristics can reflect the severity of depression that was quantified using the HAMD scale score, their correlation was analyzed in the task and the resting state, respectively. Specifically, the metric values and the HAMD score values from both the PSD and the non-PSD groups were combined into a single set. Then, the Spearman correlation was analyzed between the HAMD scores and the metric values. Figures 6, 7 illustrate the metrics that had a significant (*p* < 0.05) correlation with the HAMD scores in the task and the resting state, respectively. In both states, the nodal local efficiency (NLe), nodal clustering coefficient (NCp) and local efficiency (Eloc) had a positive correlation with the HAMD score. On the contrary, the metric degree centrality (Dc) and nodal efficiency (Ne) had a negative correlation with the HAMD score.

### 3.4. Classification

**Figure 8:**
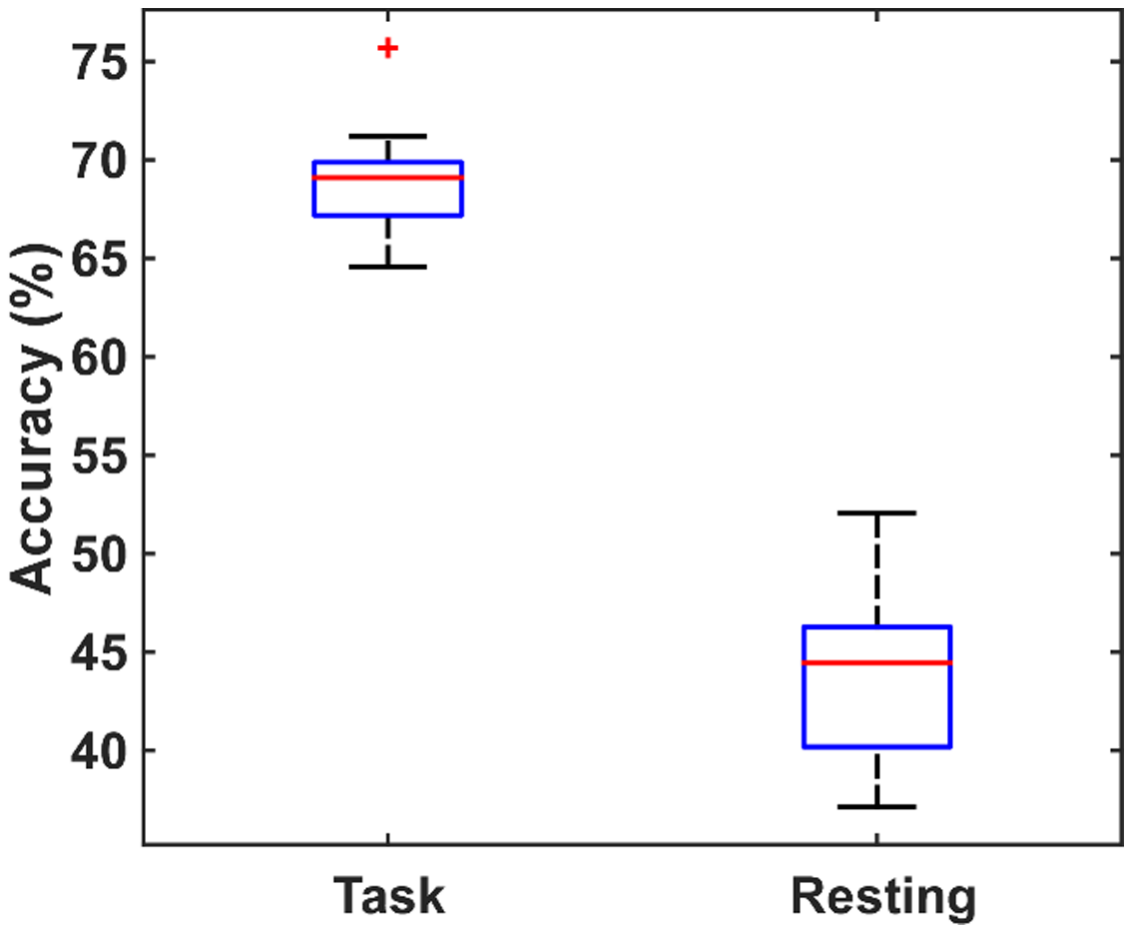
The comparison of the classification performance using the features extracted from the task state and the resting state.

In order to verify whether the brain network characteristics can serve as the biomarkers to help the diagnosis of PSD, all the 79 (9 global metrics and 5 nodal metrics for each of 14 channels) metrics were used as the features to classify between the PSD and the non-PSD patients. Then, a feature dimension reduction procedure was performed based on the principal component analysis. The eightfold cross-validation was performed and the average classification accuracy was obtained across all folds. The cross-validation procedure was repeated 10 times and the resultant accuracy using the features in the resting and the task states are compared in Figure 8. On average, the features in the task state obtained an accuracy of 69.02% ± 3.35% while the features in the resting state only obtained an accuracy of 43.94% ± 4.47%.

## 4. Discussion

In this study, in order to investigate the influence of PSD on the topology of the brain functional connectivity in stroke survivors, the fNIRS signals from the left and right DLPF cortex were collected for both the PSD and non-PSD subjects during the classic oddball cognitive task and analyzed using the graph theory. To the best of our knowledge, this is the first near-infrared brain function imaging study that targeted the brain network properties under the cognitive task instead of the resting state as previous studies for the PSD patients. Our results showed that compared with the resting state, the brain network properties were more distinct between the PSD and non-PSD patients in the task state, manifested as a larger number of connectivity and network metrics showing significant differences and a resultant better classification performance between the PSD and non-PSD patients. Specifically, in the cognitive task state, the major differences of the network topography for the PSD subjects compared with the non-PSD subjects included the decreased connectivity strength, the reduced hierarchy (b), efficiency (Eg, Ne) and betweenness centrality (Bc), and the increased assortativity (r), local efficiency (Eloc, NLe), clustering coefficient (Cp, NCp), characteristic path length (Lp) and Lambda in the small-world property, which indicated the reduced ability of the brain network to resist attacks and transmit information, and the enhanced network flexibility. Further correlation analysis showed that some of the brain network characteristics extracted from the task state were significantly correlated with the HAMD scores. More importantly, a better classification performance can be obtained using the network characteristics in the task state (69.02 ± 3.35%) as features compared with the resting state (43.94 ± 4.47%), indicating the superiority of the brain network topography in the task state to distinguish between PSD and non-PSD patients. These findings demonstrated that the brain network properties based on the graph theory under the cognitive task might provide new insights into our understanding of PSD and new methods for the diagnosis of PSD.

Ho et al. (2015) investigated the DMN of patients with severe depression in the resting state and found that the functional connectivity between the medial prefrontal cortex and both the precuneus and the cingulate gyrus were enhanced. Moreover, for the patients with PSD in the chronic phase, researchers found that the functional connectivity in the prefrontal cortex (PFC) including the left ventromedial PFC (Zhu et al., 2012; Li et al., 2013), bilateral dorsomedial PFC (Zhu et al., 2012; Li et al., 2013) and dorsolateral PFC (Li et al., 2017) increased in the resting state. Our results showed that the brain functional connectivity in the left DLPF cortex was enhanced in the PSD patients compared with the non-PSD patients in the resting state, which is consistent with previous studies. Moreover, we found that the differences in connectivity were more pronounced in the task state than in the resting state, which is consistent with the findings of Gonzalez-Castillo and Bandettini (2018) that the differences of the functional connectivity between the resting and task states are broadly distributed in the whole brain. However, unlike the increased connectivity strength in the resting state, the connectivity strength decreased in the DLPF cortex for the PSD patients in the task state. These results indicated that the PSD patients possibly had weakened capabilities for information transmission between brain regions when performing cognitive tasks, which may be related to the decline of their cognitive function.

At present, there is no unified conclusions on the brain network characteristics of depression. Zhang et al. (2018) investigated the brain network properties using the resting-state electroencephalogram (EEG) and found that compared with the healthy controls, patients with depression had a lower clustering coefficient and characteristic path length, and a higher global efficiency. In contrast, Meng et al. (2014) using the resting-state fMRI, found that compared with the healthy individuals, the global efficiency at the whole brain level of the depression patients was reduced, while the characteristic path length was increased. The inconsistence might be caused by the fact that the EEG and fMRI signals reflect the different aspects of the brain activities due to their distinct time resolutions. In this study, we found that the clustering coefficient (Cp, NCp), the characteristic path length (Lp), and the Lambda of the PSD patients were significantly higher compared with the non-PSD patients. Meng et al. (2014) found the same changing pattern of these characteristics as ours. However, the difference in Meng’s study was not significant. The possible reason was that the brain network characteristics were obtained in the task state instead of the resting state, which made the differences more obvious. It may also be cause by the fact that our research subjects were patients with PSD rather than pure depression patients. In addition, we found that the PSD patients had a higher assortative coefficient (r) and local efficiency (Eloc, NLe). Since the Sigma values were all larger than one for both the PSD and non-PSD patients in the task state (Figure 4I), it can be concluded that the brain network of both groups had small-world properties. This is consistent with the previous study that the brain network of both the depression and the healthy controls have the small-world properties (Meng et al., 2014). However, based on the variation trend of the network characteristics in the PSD patients compared with the non-PSD patients, the ability of the brain network in the PSD patients to resist attacks and integrate and transmit information was reduced. The assortativity coefficient ranging from −1 to 1 can be used to measure the network resilience (Newman, 2002). The networks with a positive assortativity coefficient are therefore likely to have a comparatively resilient core of mutually interconnected high-degree hubs (Rubinov and Sporns, 2010). If network nodes are removed from such a network or “attacked,” the entire network is more likely to be destroyed. That is, the nodes in the network with a high assortativity are more closely connected, and are more vulnerable to attacks. In this study, the PSD group has a larger assortativity coefficient, which might be one of the bases for the neuropathological damage in PSD patients. The resultant inefficient transmission of information might be another neural pathogeneses of depression. The higher clustering coefficient and local efficiency reflected the enhanced local interconnectivity of the network in the PSD patients, which provided flexibility for network disruption. This topological feature may be related to the compensation mechanism of the brain in the PSD patients. Besides, the betweenness centrality and degree centrality of the PSD patients were lower than those of the non-PSD group, indicating that the centralization degree of the PSD patients was low. For the other three nodal network characteristics (NCp, NLe and Ne), the variation trend of the PSD patients compared with the non-PSD patients was the same between the resting and the task state. However, the channels with significant differences were different.

The correlation between the brain network characteristics and the HAMD scale scores was consistent in the resting and task states. That is the local efficiency (Eloc, NLe) and the clustering coefficient (NCp) was positively correlated with the HAMD score, and the global efficiency (Ne) and the degree centrality (Dc) had a negative correlation with the HAMD scores. These significant correlations indicated that the brain network characteristics can reflect the severity of PSD to some degree.

Basically speaking, the diagnosis of a disease is to identify or classify between positive and negative individuals using some biomarkers. Although potential biomarkers of a disease are often statistically significant at the population level, the discriminatory power at the individual level is often not assessed (Arbabshirani et al., 2017). In general, it is much easier to show group differences than the predictions of individual subjects, and highly significant group differences do not always equal to a satisfactory classification performance. Therefore, in the study, we used the machine learning methods to classify between the PSD and non-PSD patients, and compared the diagnostic accuracy using the extracted brain network characteristics in the resting and task states. Our results showed that the classification accuracy reached 69.02% ± 3.35% when using the network characteristics in the task state. This performance was comparable to the average accuracy of the resting-state functional connectivity research to predict other neuropsychiatric diseases (Arbabshirani et al., 2017). As a contrast, even though the same number of brain network characteristics in the resting state was used, the classification accuracy was only 43.94% ± 4.47%. The results further demonstrated that the brain network properties in the task state can be used as the potential biomarker for the diagnosis of PSD. Even though the functional brain network analysis is not currently used for diagnosis of disease, it enables us to understand the functional connectivity of human brain from the perspective of network, and the complex network theory reveals many important topological properties of human brain structures. Brain diseases can lead to the changes in the topology of brain functional networks. With the development of research methods and theories, some scholars believe that functional brain network analysis can help assist the early diagnosis of mental diseases (Wang et al., 2021). For example, Drysdale et al. analyzed the changes of brain functional network connectivity patterns in patients with depression and divided depression into four subtypes (Drysdale et al., 2017). More recently, a review stated that functional brain network imaging has been able to facilitate early diagnosis and assist in monitoring disease progression and treatment outcomes for individual patients (Matej Perovnik et al., 2023). Therefore, it is reasonable to predict that the brain functional network analysis can potentially help with the early diagnosis of brain diseases in the near future.

In this study, there were no significant differences in the age, gender, time after onset, education level, stroke type, lesion location, MMSE, and MoCA scores between the two groups, which demonstrated the homogeneity between the two groups and therefore improved the reliability of our conclusions. Although the two groups of stroke patients showed the similar degrees of brain injury, the non-PSD patients did not show the depression symptoms. This suggests that the brain injury degree may not be the determinant in the pathogenesis of PSD. Instead, other factors might be of more significance, which is consistent with recent studies (Gong and He, 2015). A recent systematic review and meta-analysis (Lu Liu et al., 2023) showed that the pooled prevalence of PSD is 27% (95% CI 25–30) at any time point after stroke, and stroke survivors with early-onset depression (within 3 months after stroke) are at high risks for remaining depressed and make up two-thirds of the incident cases during 1 year after stroke. Therefore, the inclusion criteria in this study were set to 1–12 months after stroke onset. However, the poststroke time of the recruited subjects in the end was 32.25–97.5 (Q1–Q3) days (Table 1) for the PSD subjects. This was consistent with the study showing that the pooled cumulative incidence within 1 year was 38% (95% CI 33–43), and the majority [71% (95% CI 65–76)] of cases of depression had onset during the first 3 months after stroke (Lu Liu et al., 2023). Although different clinical studies have shown the complexity of PSD prevalence and distinct trajectories of PSD for individuals, there are no studies ever tracking the brain network properties longitudinally for individual PSD patients. This might help improve our understanding of the underlying pathogenesis of PSD and would be conducted in our further study.

There were several limitations in this study. Firstly, the fNIRS signals were only collected from the dorsolateral prefrontal cortex. Although the dorsolateral prefrontal cortex is the most investigated brain area for PSD, the ventromedial prefrontal cortex, anterior cingulate gyrus, posterior cingulate gyrus/precuneus, amygdala, caudate nucleus, hippocampus, and other regions are also associated with depression. Secondly, the classification of patients in terms of lesion location is relatively rough with only two groups, i.e., lesion in cortex or subcortex. Futures studies will recruit more stroke survivors with different lesion locations and investigate the brain network properties at the whole brain level in the task state, in order to further explore the possible neural mechanism of PSD in terms of altered brain network properties.

## 5. Conclusion

In this study, the brain network characteristics of PSD patients in both the cognitive task and the resting state were extracted using the fNIRS signals and compared with the network characteristics of non-PSD patients. The results showed that the differences of the network characteristics between the PSD and non-PSD patients were more distinct in the task state rather than the resting state. The altered brain network properties of the PSD patients demonstrated the reduced ability of the brain network to resist attacks and transmit information, and the enhanced network flexibility. The results of the classification between PSD and non-PSD patients further demonstrated the superiority of the network characteristics extracted in the task state on revealing the altered topography of the brain functional connectivity due to PSD. These findings demonstrated the feasibility and superiority of the brain network topography in the task state to explore the neural mechanism of PSD, which provides new insights into our understanding of PSD and new methods for the diagnosis of PSD.

## Data availability statement

The raw data supporting the conclusions of this article will be made available by the authors, without undue reservation.

## Ethics statement

The studies involving humans were approved by the Ethics Committee of the First Affiliated Hospital of Xi’an Jiaotong University. The studies were conducted in accordance with the local legislation and institutional requirements. The participants provided their written informed consent to participate in this study.

## Author contributions

YP and YZ conceptualized the study. JG, ZY, CF, JD, and SS performed the experiment. YZ and CL analyzed the data. YP, YZ, JQ, and JW drafted the manuscript. All authors revised the manuscript and approved the final version.

## Funding

This work was supported by the STI 2030—Major Projects (grant no. 2022ZD0209800), the Qin Chuang Yuan Talent Project (grant no. QCYRCXM-2022-34), and the Natural Science Foundation of Shaanxi Province (grant no. 2021JM-279).

## Conflict of interest

The authors declare that the research was conducted in the absence of any commercial or financial relationships that could be construed as a potential conflict of interest.

## Publisher’s note

All claims expressed in this article are solely those of the authors and do not necessarily represent those of their affiliated organizations, or those of the publisher, the editors and the reviewers. Any product that may be evaluated in this article, or claim that may be made by its manufacturer, is not guaranteed or endorsed by the publisher.
